# EEG‐Based Cortical Alterations in Patients Following Total Hip and Knee Arthroplasty Due to Osteoarthritis: Pre‐ and Post‐Operative Assessment

**DOI:** 10.1002/brb3.71464

**Published:** 2026-04-29

**Authors:** Pawel Piotr Dobrakowski, Jaroslaw Szyszka

**Affiliations:** ^1^ Psychology Institute Humanitas Academy In Sosnowiec Sosnowiec Poland; ^2^ Orthopedic Surgery Department Opole Rehabilitation Center Korfantów Poland

**Keywords:** arthroplasty, electroencephalography (EEG), motor imagery, neuroplasticity, osteoarthritis, quantitative electroencephalography (qEEG)

## Abstract

Quantitative electroencephalography (qEEG) has not yet been systematically applied to characterize cortical bioelectrical activity in patients undergoing total hip or knee arthroplasty.

The aim of this study was to evaluate pre‑ to post‑operative changes in cortical activity and their relationship with functional outcomes after arthroplasty.

Neuroplastic changes were examined in 81 patients with end‑stage osteoarthritis (33 hip, 48 knee) scheduled for primary total joint arthroplasty. All participants underwent clinical assessment one week before surgery and three months postoperatively using the Harris hip score (HHS), knee society score (KSS), and oxford knee score (OKS), together with full‑cap EEG recordings (19 electrodes) during resting state and stair‑climbing motor imagery.

EEG analysis revealed a generalized increase in low alpha band power during rest after arthroplasty in both hip (*n* = 33) and knee (*n* = 48) patients, particularly over frontocentral and parietal regions (mean increases ∼1–3 µV^2^; *p* < 0.001; Cohen's d up to 1.6). High alpha band changes were less consistent and often moved in the opposite direction over posterior sites, especially after knee arthroplasty and during motor imagery (*p* < 0.001). Theta power also increased post‑surgery, most prominently over frontal and central electrodes during both rest and motor imagery (*p* < 0.001). Only a few moderate correlations between changes in alpha band power and improvements in joint‐specific clinical scores reached significance (Spearman's ρ, *p* < 0.05).

These findings indicate that functional recovery after total hip and knee replacement is accompanied by measurable, band‑specific reorganization of cortical oscillatory activity, with partially distinct patterns between joints. The results support a relevant contribution of central mechanisms to postoperative outcomes and highlight qEEG as a promising tool for monitoring neuroplastic adaptation following major joint arthroplasty.This study reveals significant neuroplastic changes in cortical EEG activity post total hip/knee arthroplasty in osteoarthritis patients, with increased low alpha and theta power over frontocentral/parietal regions during rest and motor imagery. These alterations accompany functional improvements (HHS/KSS), suggesting qEEG's potential for monitoring recovery.

## Graphical abstract

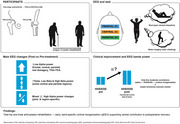

## 1 Introduction

Osteoarthritis is one of the most prevalent joint disorders globally. In 2020, it was estimated that approximately 595 million individuals were affected worldwide, accounting for 7.6% of the global population—a figure that reflects an increase of more than 130% in total cases since 1990 (GBD 2021 Osteoarthritis Collaborators 2023).

In Poland, 64,694 hip arthroplasties and 37,821 knee arthroplasties were performed in 2022. Women accounted for 56% of hip procedures and 69% of knee procedures. Over 80% of these interventions were carried out in patients aged 60–79, which correlates with the progressive nature of osteoarthritis, notably the increase in pain with advancing age (Narodowy Fundusz Zdrowia 2025).

Cutaneous mechanoreceptors and joint proprioceptors, in conjunction with the vestibular system and visual apparatus, serve as afferent sources of information regarding body position and balance for the central nervous system (CNS). Additionally, these pathways transmit nociceptive signals and mediate reflex arcs. Afferent stimuli are processed at three hierarchical CNS levels—spinal cord, brainstem, and motor cortex—supporting motor planning, unconscious joint stabilization, as well as postural and balance control (Riemann and Lephart 2002). Proprioceptive acuity is integral to maintaining knee joint stability and coordinating the motor system to ensure dynamic postural control and balance (Röijezon et al. 2015). It has been suggested that deafferentation may be difficult to reverse, both due to altered input from the knee joint and biomechanical stabilisation following surgical reconstruction (Giesche et al. 2022).

Intervention within a major lower limb joint involving disruption of the extensive receptor zone surrounding the joint must be regarded not only as a peripheral injury but also as a complex process with neurophysiological consequences. This interference may induce nonspecific alterations in cortical bioelectrical activity, not necessarily confined to direct injury outcomes, which have been well‐documented in studies concerning the anterior cruciate ligament (ACL) (Baumeister et al. 2008; Baumeister et al. 2011; Neto et al. 2019; Piskin et al. 2025). Furthermore, unilateral knee osteoarthritis has been shown to disrupt proprioceptive function not only in the affected limb but also in the other limb (Knoop et al. 2011).

Accumulating neuroimaging and neurophysiological evidence indicates that ACL injury is associated with substantial alterations within the CNS. Scoping and conceptual reviews have demonstrated consistent changes in activation and connectivity within primary sensorimotor, premotor, and cerebellar networks after ACL injury or reconstruction, interpreted as compensatory reorganization in response to disrupted ligamentous proprioception and increased sensory “noise” in afferent input. Together, these findings support the view that a major perturbation of joint‐related sensory feedback can induce central sensorimotor reorganization rather than remaining a purely peripheral problem. By analogy, end‑stage osteoarthritis of the hip or knee, followed by total joint arthroplasty and rehabilitation, also profoundly alters the quantity and quality of afferent information from the operated joint (Neto et al. 2019; Grooms et al. 2015; Schnittjer et al. 2025). In the present study, we therefore hypothesized that these peripheral changes would be accompanied by measurable modifications in cortical bioelectrical activity, as assessed by EEG at rest and during motor imagery.

Nevertheless, patient satisfaction levels following arthroplasty vary significantly between joint types. Individuals undergoing hip replacement often report higher satisfaction compared to those who receive knee replacement. This disparity may stem from the near‐anatomical restoration of the hip joint function. The replaced joint moves within an identical range to that the biological joint prior to the disease process. This is not the case for knee prostheses. Bourne reported a dissatisfaction rate of 19% among knee replacement patients, while Noble documented a comparable rate of 14% (Bourne et al. 2010; Noble et al. 2006). This is particularly disappointing given that these statistics pertain to patients free of implant‐related complications or identifiable anatomical abnormalities. This also occurs in some patients whose range of motion and muscle strength are assessed as very good. This may suggest that additional components are involved, particularly central or cognitive factors.

Some patients continue to consciously monitor their prosthetic joint, particularly when it behaves incongruently with their expectations based on the former biological joint during functional tasks (Loth et al. 2018). These individuals are more vigilant and tend to overprotect the replaced knee joint. They hesitate when engaging in exercises or physical risks (Stenquist et al. 2015; Webster et al. 2015; Woolhead et al. 2005). As such, there is growing recognition of the need to address kinesiophobia—defined here as a fear of fully weight‐bearing and trusting the operated limb. Among patients undergoing knee replacement, previous studies have demonstrated a negative correlation between high levels of kinesiophobia and joint range of motion, gait performance, and pain thresholds (Güney‐Deniz et al. 2017; Doury‐Panchout et al. 2015). Meanwhile, early postoperative functional outcomes following hip replacement do not appear to show a similar correlation with kinesiophobia (Morri et al. 2020).

Following joint replacement surgery, patients typically undergo rehabilitation. This is designed to prevent implant destabilisation, restore functional muscle strength and tone, reduce thromboembolic risk, facilitate verticalization and gait retraining, achieve near‐physiological range of motion, and mitigate the development of contractures and pain syndromes (Gremeaux et al. 2008; Rahmann et al. 2009; Stockton and Mengersen 2009).

The surgical act of arthroplasty, the restoration of joint mobility through rehabilitation, and the alteration in sensory input from the operated joint to the CNS are likely to modulate cortical bioelectrical activity, as captured by electroencephalography (EEG). Particular attention has been given to specific EEG frequency bands relevant to sensorimotor function: delta waves (0.5–4 Hz), associated with conscious attention and cortical integration (i.e., the convergence of multimodal sensory input to shape motor responses) (Bruns and Eckhorn 2004); theta waves (4–7 Hz), related to memory processes, including working memory (short‐term memory required to determine action based on perception) (Sauseng et al. 2010); and low (8–10 Hz) and high alpha waves (11–13 Hz), implicated in cortical deactivation and inhibition, particularly in relation to sensorimotor activity (Baumeister et al. 2008).

The aim of this study was to evaluate changes in EEG parameters in patients with hip or knee osteoarthritis before and after total joint arthroplasty, and to relate these changes to functional outcomes. We further sought to identify potential shifts in cortical bioelectrical activity during both passive rest and motor imagery following the surgical intervention.

## 2 Materials and Methods

### 2.1 Participants

The study included patients treated at the Opole Rehabilitation Center, Korfantów, who underwent knee or hip arthroplasty at the Department of Orthopaedic Surgery between 2020 and 2021 and subsequently completed a standard 21‐day rehabilitation program at the Department of Rehabilitation.

86 patients entered the study, and 81 completed all stages; 5 patients (3 hip, 2 knee) were lost to follow‐up before the postoperative assessment. Their characteristics are presented in Table 1.

**TABLE 1:** Characteristics of the patients who completed all study stages (*n* = 81), stratified by joint, age, sex, and side. Hip = hip arthroplasty group, knee = knee arthroplasty group, F = female, M = male, R = right, L = left.

—	Sex	Age	Side
Hip	22 F/11 M	62.4 ± 8.5	24 R/9 L
Knee	35 F/13 M	66.0 ± 6.4	27 R/21 L

Inclusion criteria were:

- Radiographically confirmed primary hip or knee osteoarthritis (Kellgren–Lawrence grade II–IV) with clinical symptoms consistent with osteoarthritis.
- Qualification for primary total joint arthroplasty at Opole Rehabilitation Center, Korfantów.
- Willingness to undergo preoperative assessment and 3‐month follow‐up after surgery, including a standard 21‐day rehabilitation program.

Exclusion criteria were:

- History of central nervous system disease, prior neurosurgical procedures, or current pharmacological treatment known to substantially affect cortical bioelectrical activity.
- Self‐reported left‐limb dominance, to enhance sample homogeneity.
- Age >75 years, due to age‐related alterations in EEG signal characteristics.

### 2.2 Study Protocol

Clinical and electrophysiological assessments (EEG) were performed one week prior to arthroplasty. All participants underwent standard inpatient postoperative rehabilitation lasting approximately three weeks. Follow‑up clinical and EEG assessments were conducted three months after surgery.

All surgical procedures were performed by the same orthopedic surgeon (study author) to ensure procedural consistency. Total hip arthroplasty (THA) was conducted via a posterolateral approach using cementless components with either metal‑on‑polyethylene or ceramic‑on‑polyethylene articulation and femoral head diameters of 32 mm or 36 mm. Total knee arthroplasty (TKA) employed a medial parapatellar approach with posterior cruciate ligament preservation, cemented implants, and a fixed‑bearing polyethylene insert; the patellar component was not resurfaced.

Written informed consent was obtained from each participant prior to inclusion in the study. The research protocol received ethical approval from the Bioethics Committee of the Academy of Physical Education in Katowice (Resolution No. 1/2020, dated July 9, 2020).

### 2.3 Clinical Scales

The efficacy of the surgical intervention was evaluated using the knee society score (KSS) and Harris hip score (HHS), which were administered in person by the study authors, all of whom were trained physicians. Self‐reported outcomes were assessed with the oxford knee score (OKS), with questionnaire data obtained directly by the authors during the follow‐up hospital visit (Harris 1969; Dawson et al. 1998; Insall et al. 1989).

### 2.4 EEG Analysis

A 19‐channel EEG (10–20 system; TruScan EEG, Deymed Diagnostic 2019) recorded cortical activity at rest and during stair‐climbing motor imagery. Sampling: 1024 Hz; filters: 50 Hz notch, 1–40 Hz bandpass; impedance <5 kΩ (bilateral pairs <1 kΩ); reference: earlobe.

ROIs comprised frontal (F3, Fz, F4), central (C3, Cz, C4), and parietal (P3, Pz, P4) electrodes, consistent with recent literature (Gebel et al. 2022; Busch et al. 2024).

Power spectral density (PSD) was computed via FFT using EEGSTATS (EEGLAB/MATLAB), analyzing theta (4–7.99 Hz), low alpha (8–9.99 Hz), high alpha (10–12.99 Hz), low beta (13–17.99 Hz), and high beta (18–30 Hz) bands.

**FIGURE 1:**
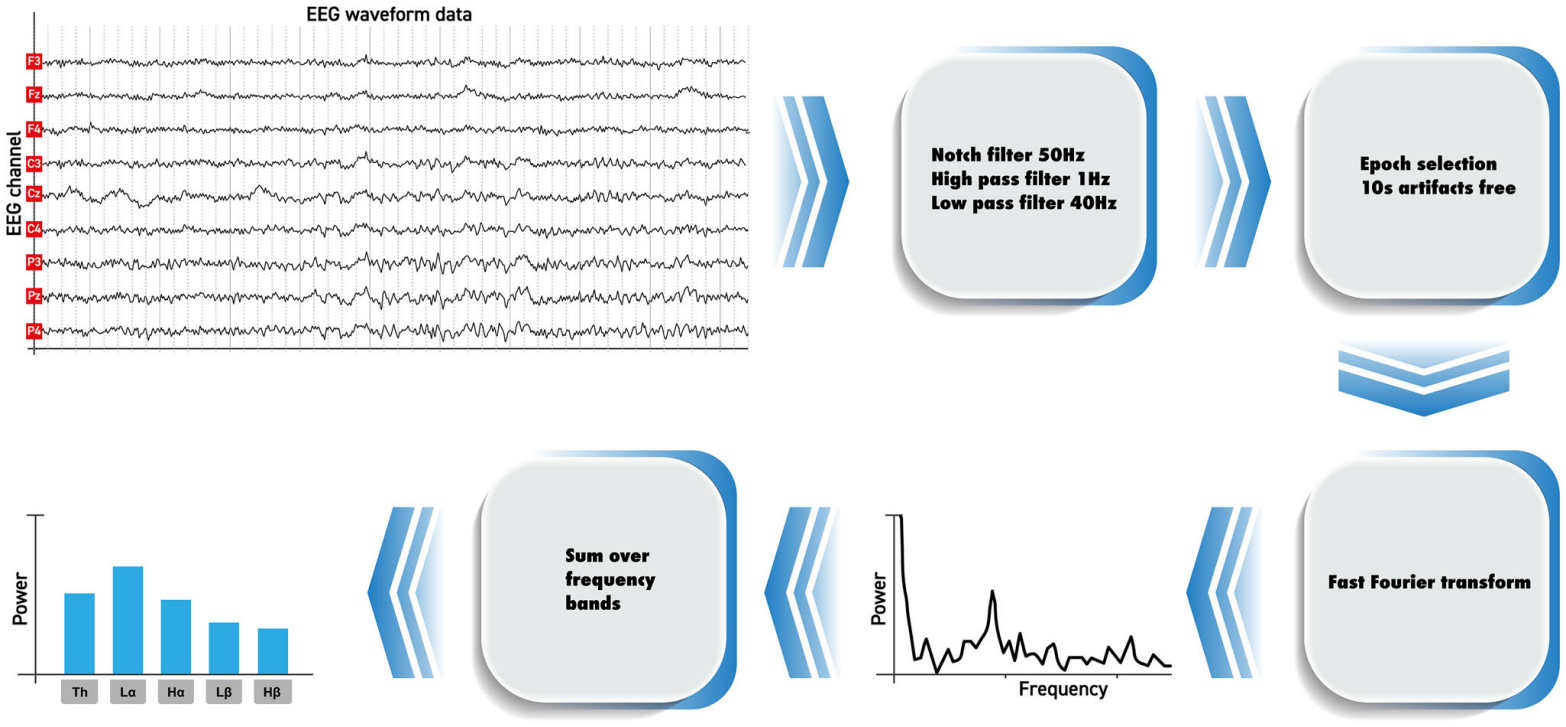
EEG processing pipeline; EEG data is imported, band pass filtered between 1 and 40 Hz, epoch selection of ROI electrode channels, converted to spectral data via Fourier transform and summed over theta, low alpha, high alpha low beta, and high beta bands, respectively.

Peak alpha frequency (PAF; 7–15 Hz) averaged 9.27 ± 0.87 Hz (low alpha range) in 69/81 participants (85%), justifying alpha subdivision (Dobrakowski and Łebecka 2020; Park et al. 2024; Klimesch 2012). Low‐voltage recordings in the remaining cases showed poor spatial differentiation (see Figure 1).

### 2.5 EEG Recording

A 20 min EEG was recorded with participants lying supine and eyes closed. Photostimulation and hyperventilation were applied to exclude latent seizure activity.

Before recording, participants received standardized instructions for two tasks: (1) resting state—imagining complete relaxation following the verbal cue “Rest now”; and (2) motor imagery—imagining stair climbing after the cue “Now begin imagining climbing stairs.” Each condition lasted one minute, followed by the cue “Return to your normal state.” A brief practice ensured comprehension and comfort.

The initial two minutes served for habituation and were excluded from analysis. EEG segments followed this sequence: rest (2 min), motor imagery (6 min), photostimulation (6–40 Hz; 9 min), and hyperventilation (13 min for 3 min).

Segments with drowsiness or stimulation artefacts were omitted. For each participant, one artefact‑free 10 s epoch per condition (rest and motor imagery) was selected based on signal quality and channel integrity. The first usable epoch was analyzed to minimize non‑stationarity and reduce multiple comparisons.

### 2.6 Statistical Analysis

Analyses were performed in MATLAB (MathWorks). Data are reported as means ± SD. Normality was assessed via the Jarque–Bera test (skewness/kurtosis).

Paired comparisons used Student's t‐test for normally distributed data and Wilcoxon signed‐rank test otherwise (p < 0.05). Effect sizes were Cohen's *d* = (post‐pre mean difference)/SD of differences for t‐tests, and r = |Z|/√N for Wilcoxon tests (N = observations). Interpretation thresholds:

- Cohen's d: 0.2 (small), 0.5 (medium), ≥0.8 (large).
- Wilcoxon r: 0.1 (small), 0.3 (medium), ≥0.5 (large).

Spearman's ρ correlations used thresholds for |ρ|: <0.2 (none), 0.2–0.4 (weak), 0.4–0.7 (moderate), 0.7–0.9 (strong), >0.9 (very strong).

## 3 Results

### 3.1 Patient Characteristics

Significant postoperative improvements in clinical scores were observed in both groups (KSS in the knee cohort, HHS in the hip cohort). Self‐reported outcomes further corroborated postoperative improvements, as reflected in the OKS, which increased significantly from 18.50 ± 3.39 preoperatively to 41.28 ± 5.98 postoperatively. We did not further analyze the OKS data, as there is no certified, validated equivalent for the hip in Polish. Data are presented in Tables 2 and 3 for clarity.

**TABLE 2:** Change in the HHS scale post hip arthroplasty.

—	Before treatment (*n* = 36)	Post treatment (*n* = 33)	—	—
HHS	Mean ± SD	Mean ± SD	*Z*	*p*
Total score	45.14 ± 14.79	85.03 ± 6.16	−7.02	<0.001

**TABLE 3:** Change in the KSS scale post knee arthroplasty.

—	Before treatment (*n* = 50)	Post treatment (*n* = 48)	—	—
KSS	Mean ± SD	Mean ± SD	*Z*	*p*
Knee score	41 ± 15.51	86.36 ± 12.29	−8.50	<0.001
Functional scale	51.5 ± 12.59	89.09 ± 9.44	−8.52	<0.001

### 3.2 Changes in EEG Absolute Band Power (Post‐ vs Pre‐Treatment)

**FIGURE 2:**
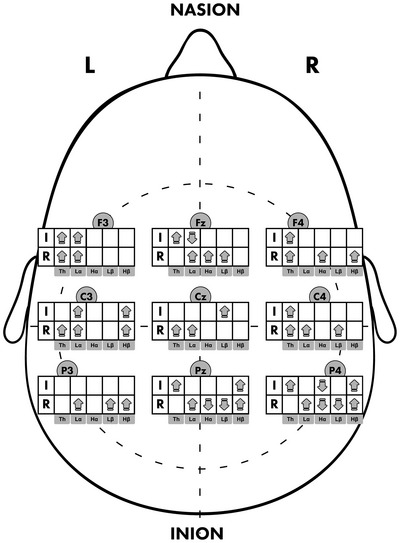
Hip. Arrows up indicate an increase, arrows down indicate a decrease in EEG band power after the procedure if *p* < 0.05, and *r* > 0.5 or *d*_z_ > 0.8. I = motor imagery; and R = rest.

**FIGURE 3:**
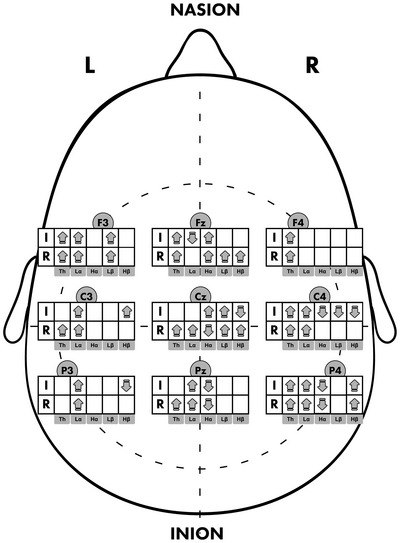
Knee. Arrows up indicate an increase, arrows down indicate a decrease in EEG band power after the procedure if *p* < 0.05, and *r* > 0.5 or *d*_z_ > 0.8. I = motor imagery; and R = rest.

Statistically significant (*p* < 0.05) changes in absolute power were observed across most electrodes during rest and motor imagery, often with large effects (*r* > 0.5; *d*_z_ > 0.8), particularly in low alpha. These robust changes suggest clinically relevant neuroplasticity, warranting further validation. The data are presented for clarity in Tables 4, 5, 6 and 7 and Figures 2, 3.

**TABLE 4:** Arithmetic means of change in EEG bands power for patients in the hip arthroplasty group (motor imagery): Difference (post–re arthroplasty). A positive value indicates an increase, a negative value indicates a decrease of absolute power after the procedure. pre tr. = before arthroplasty, post tr. = after arthroplasty, diff. = difference, *d*_z_ = Cohen's effect size, *r* = Wilcoxon effect size.

µV^2^	—	Theta	Low alpha	High alpha	Low beta	High beta
F3	Mean ± SD pre tr.	3.504 ± 0.881	6.997 ± 1.828	4.090 ± 1.146	2.448 ± 0.939	2.289 ± 0.575
—	Mean ± SD post tr.	4.539 ± 1.602	8.964 ± 3.358	4.034 ± 1.071	2.501 ± 0.883	2.419 ± 0.734
—	Post‐ pre diff. ± SD	1.035 ± 1.217	1.968 ± 2.892	−0.056 ± 0.742	0.053 ± 0.269	0.130 ± 0.353
—	diff. *p*‐value	<0.001	0.001	0.795	0.032	0.143
—	diff. effect size	0.823 (*r*)	0.569 (*r*)	0.045 (*r*)	0.374 (*r*)	0.367 (*d*_z_)
Fz	Mean ± SD pre tr.	2.751 ± 0.673	9.195 ± 2.469	8.277 ± 2.413	2.321 ± 0.874	2.085 ± 0.552
—	Mean ± SD post tr.	3.403 ± 1.499	7.807 ± 3.262	8.603 ± 3.592	2.218 ± 0.818	2.173 ± 1.164
—	Post‐ pre diff. ± SD	0.652 ± 1.267	−1.388 ± 2.353	0.326 ± 3.294	−0.103 ± 0.482	0.088 ± 0.976
—	diff. *p*‐value	<0.001	0.001	0.013	0.044	0.083
—	diff. effect size	0.725 (*r*)	0.580 (*r*)	0.434 (*r*)	0.351 (*r*)	0.302 (*r*)
F4	Mean ± SD pre tr.	4.103 ± 1.168	8.489 ± 2.562	4.876 ± 1.124	2.375 ± 0.847	2.276 ± 0.705
—	Mean ± SD post tr.	4.858 ± 1.642	8.649 ± 2.885	5.063 ± 1.658	2.388 ± 0.870	2.722 ± 1.687
—	Post‐ pre diff. ± SD	0.755 ± 0.986	0.160 ± 1.938	0.188 ± 1.399	0.013 ± 0.386	0.446 ± 1.451
—	diff. p‐value	<0.001	0.084	0.006	0.816	0.720
—	diff. effect size	0.642 (*r*)	0.399 (*r*)	0.478 (*r*)	0.040 (*r*)	0.062 (*r*)
C3	Mean ± SD pre tr.	6.608 ± 2.149	7.053 ± 2.173	7.302 ± 1.661	2.279 ± 0.892	2.084 ± 0.582
—	Mean ± SD post tr.	6.671 ± 2.226	9.685 ± 3.138	7.890 ± 2.665	2.407 ± 0.962	2.735 ± 1.220
—	Post‐ pre diff. ± SD	0.062 ± 1.458	2.633 ± 1.670	0.588 ± 2.464	0.127 ± 0.546	0.651 ± 1.127
—	diff. *p*‐value	0.126	<0.001	0.018	0.768	<0.001
—	diff. effect size	0.387 (*r*)	1.577 (*d*_z_)	0.412 (*r*)	0.051 (*r*)	0.689 (*r*)
Cz	Mean ± SD pre tr.	3.959 ± 1.152	8.487 ± 2.683	5.875 ± 1.424	2.177 ± 0.792	1.955 ± 0.485
—	Mean ± SD post tr.	4.422 ± 2.366	9.629 ± 3.483	6.837 ± 2.228	2.556 ± 0.991	2.194 ± 1.189
—	Post‐ pre diff. ± SD	0.463 ± 1.948	1.142 ± 2.373	0.962 ± 1.996	0.379 ± 0.479	0.239 ± 1.075
—	diff. *p*‐value	0.044	0.009	0.007	<0.001	0.198
—	diff. effect size	0.350 (*r*)	0.581 (*d*_z_)	0.369 (*r*)	0.605 (*r*)	0.224 (*r*)
C4	Mean ± SD pre tr.	3.084 ± 1.071	9.049 ± 3.174	5.877 ± 2.287	2.098 ± 0.717	2.198 ± 1.294
—	Mean ± SD post tr.	4.696 ± 2.663	9.421 ± 3.097	5.899 ± 1.865	2.120 ± 0.788	2.162 ± 1.379
—	Post‐ pre diff. ± SD	1.612 ± 2.277	0.372 ± 2.141	0.022 ± 2.219	0.022 ± 0.409	−0.035 ± 0.559
—	diff. *p*‐value	<0.001	0.026	0.549	0.648	0.094
—	diff. effect size	0.869 (*r*)	0.387 (*r*)	0.104 (*r*)	0.079 (*r*)	0.452 (*r*)
P3	Mean ± SD pre tr.	4.714 ± 2.308	10.018 ± 3.562	9.925 ± 3.286	1.728 ± 0.651	1.956 ± 0.989
—	Mean ± SD post tr.	4.735 ± 2.921	11.061 ± 3.420	11.003 ± 4.449	1.753 ± 0.608	1.982 ± 0.959
—	Post‐ pre diff. ± SD	0.022 ± 1.054	1.043 ± 1.450	1.078 ± 2.507	0.024 ± 0.323	0.026 ± 0.367
—	diff. *p*‐value	0.075	<0.001	0.019	0.191	0.686
—	diff. effect size	0.310 (*r*)	0.719 (*d*_z_)	0.530 (*d*_z)_	0.227 (*r*)	0.071 (*d*_z_)
Pz	Mean ± SD pre tr.	3.956 ± 2.311	10.247 ± 3.659	11.472 ± 3.877	2.756 ± 1.180	1.837 ± 0.898
—	Mean ± SD post tr.	4.580 ± 2.482	11.560 ± 3.882	10.928 ± 3.949	3.853 ± 3.546	2.061 ± 0.924
—	Post‐ pre diff. ± SD	0.624 ± 0.445	1.313 ± 2.497	−0.545 ± 1.690	1.098 ± 3.538	0.224 ± 0.258
—	diff. *p*‐value	<0.001	0.005	0.101	0.543	<0.001
—	diff. effect size	1.402 (*d*_z_)	0.526 (*d*_z_)	0.670 (*r*)	0.106 (*r*)	0.742 (*r*)
P4	Mean ± SD pre tr.	4.029 ± 2.300	10.416 ± 3.792	12.839 ± 4.325	2.871 ± 1.188	1.828 ± 0.824
—	Mean ± SD post tr.	4.773 ± 2.534	10.543 ± 3.535	11.492 ± 4.091	2.885 ± 1.120	2.301 ± 0.923
—	Post‐ pre diff. ± SD	0.744 ± 0.854	0.128 ± 1.712	−1.348 ± 1.928	0.015 ± 0.439	0.473 ± 0.242
—	diff. *p*‐value	<0.001	0.118	<0.001	0.362	<0.001
—	diff. effect size	0.792 (*r*)	0.272 (*r*)	0.655 (*r*)	0.159 (*r*)	1.957 (*d*_z_)

**TABLE 5:** Arithmetic means of change in EEG bands power for patients in the hip arthroplasty group (rest): Difference (post–pre arthroplasty). A positive value indicates an increase, a negative value indicates a decrease of absolute power after the procedure.

µV^2^	—	Theta	Low alpha	High alpha	Low beta	High beta
F3	Mean ± SD pre tr.	2.568 ± 0.619	7.753 ± 2.290	3.514 ± 1.028	2.505 ± 0.960	2.478 ± 0.657
—	Mean ± SD post tr.	3.349 ± 0.829	9.015 ± 3.333	3.602 ± 0.901	2.562 ± 0.946	2.453 ± 0.731
—	Post‐ pre diff. ± SD	0.781 ± 0.540	1.262 ± 2.059	0.088 ± 0.551	0.057 ± 0.347	−0.025 ± 0.244
—	diff. *p*‐value	<0.001	<0.001	0.520	0.351	0.563
—	diff. effect size	1.447 (*d*_z_)	0.653 (*r*)	0.112 (*r*)	0.165 (*d*_z_)	0.102 (*d*_z_)
Fz	Mean ± SD pre tr.	2.615 ± 0.594	5.348 ± 1.287	3.675 ± 0.927	2.155 ± 0.790	2.207 ± 0.615
—	Mean ± SD post tr.	2.701 ± 0.775	7.876 ± 2.581	4.743 ± 1.224	2.582 ± 1.089	2.464 ± 1.055
—	Post‐ pre diff. ± SD	0.086 ± 0.471	2.527 ± 2.255	1.068 ± 0.693	0.427 ± 0.594	0.257 ± 0.757
—	diff. *p*‐value	0.002	<0.001	<0.001	<0.001	0.023
—	diff. effect size	0.257 (*r*)	0.788 (*r*)	1.542 (*d*_z_)	0.860 (*r*)	0.397 (*r*)
F4	Mean ± SD pre tr.	3.930 ± 1.009	5.254 ± 1.665	4.402 ± 1.013	2.245 ± 0.751	1.958 ± 0.686
—	Mean ± SD post tr.	4.810 ± 1.475	5.352 ± 1.495	4.921 ± 1.614	2.458 ± 0.923	2.279 ± 0.883
—	Post‐ pre diff. ± SD	0.880 ± 1.274	0.098 ± 0.631	0.519 ± 1.236	0.214 ± 0.529	0.321 ± 0.610
—	diff. *p*‐value	<0.001	0.062	0.003	0.189	<0.001
—	diff. effect size	0.692 (*r*)	0.325 (*r*)	0.509 (*r*)	0.229 (*r*)	0.676 (*r*)
C3	Mean ± SD pre tr.	4.885 ± 1.759	7.732 ± 3.232	6.641 ± 1.509	2.494 ± 1.043	2.348 ± 0.569
—	Mean ± SD post tr.	6.066 ± 2.467	9.196 ± 2.925	7.599 ± 2.485	2.499 ± 1.178	2.578 ± 0.763
—	Post‐ pre diff. ± SD	1.181 ± 1.363	1.464 ± 2.827	0.958 ± 2.196	0.005 ± 0.345	0.230 ± 0.436
—	diff. *p*‐value	<0.001	<0.001	0.011	0.940	0.001
—	diff. effect size	0.869 (*r*)	0.642 (*r*)	0.442 (*r*)	0.013 (*d*_z_)	0.565 (*r*)
Cz	Mean ± SD pre tr.	4.047 ± 1.225	9.042 ± 2.629	6.556 ± 1.448	2.333 ± 0.855	2.282 ± 0.588
—	Mean ± SD post tr.	5.099 ± 1.728	10.844 ± 3.896	6.532 ± 1.591	2.577 ± 0.999	2.465 ± 0.889
—	Post‐ pre diff. ± SD	1.053 ± 1.095	1.802 ± 2.276	−0.024 ± 1.194	0.244 ± 0.587	0.183 ± 0.756
—	diff. *p*‐value	<0.001	<0.001	0.910	0.153	0.173
—	diff. effect size	0.807 (*r*)	0.717 (*r*)	0.020 (*d*_z_)	0.249 (*r*)	0.243 (*d*_z_)
C4	Mean ± SD pre tr.	3.928 ± 1.182	8.774 ± 2.931	6.699 ± 1.823	2.260 ± 0.799	2.244 ± 0.548
—	Mean ± SD post tr.	4.979 ± 1.790	12.312 ± 7.308	6.375 ± 1.591	2.455 ± 0.906	2.302 ± 0.553
—	Post‐ pre diff. ± SD	1.051 ± 1.217	3.538 ± 5.184	−0.325 ± 1.745	0.194 ± 0.432	0.058 ± 0.272
—	diff. *p*‐value	<0.001	<0.001	0.688	0.003	0.230
—	diff. effect size	0.818 (*r*)	0.860 (*d*_z_)	0.070 (*r*)	0.515 (*r*)	0.213 (*r*)
P3	Mean ± SD pre tr.	4.409 ± 1.525	10.510 ± 3.729	10.259 ± 2.459	1.710 ± 0.731	1.329 ± 0.323
—	Mean ± SD post tr.	4.218 ± 1.373	12.548 ± 3.713	10.772 ± 3.249	1.850 ± 0.633	1.721 ± 0.469
—	Post‐ pre diff. ± SD	−0.191 ± 0.637	2.039 ± 2.149	0.512 ± 2.649	0.140 ± 0.268	0.392 ± 0.375
—	diff. *p*‐value	0.260	<0.001	0.091	<0.001	<0.001
—	diff. effect size	0.196 (*d*_z_)	0.703 (*r*)	0.294 (*r*)	0.630 (*r*)	0.946 (*r*)
Pz	Mean ± SD pre tr.	3.535 ± 1.212	10.420 ± 3.794	11.632 ± 2.614	2.601 ± 1.151	1.865 ± 0.487
—	Mean ± SD post tr.	3.701 ± 1.171	12.336 ± 7.150	9.852 ± 3.393	2.439 ± 0.981	8.730 ± 5.518
—	Post‐ pre diff. ± SD	0.166 ± 0.612	1.916 ± 5.961	−1.780 ± 2.679	−0.162 ± 0.372	6.865 ± 5.299
—	diff. *p*‐value	0.586	0.002	<0.001	0.001	<0.001
—	diff. effect size	0.095 (*d*_z_)	0.549 (*r*)	0.670 (*r*)	0.604 (*r*)	1.296 (*r*)
P4	Mean ± SD pre tr.	3.439 ± 0.960	10.639 ± 3.747	12.262 ± 2.705	3.089 ± 1.201	1.872 ± 0.468
—	Mean ± SD post tr.	3.446 ± 1.080	12.234 ± 3.876	9.788 ± 3.037	2.815 ± 1.098	2.279 ± 0.786
—	Post‐ pre diff. ± SD	0.007 ± 0.633	1.595 ± 1.908	−2.474 ± 2.534	−0.273 ± 0.388	0.407 ± 0.629
—	diff. *p*‐value	0.108	<0.001	<0.001	<0.001	<0.001
—	diff. effect size	0.280 (*r*)	0.726 (*r*)	0.658 (*r*)	0.613 (*r*)	0.790 (*r*)

Abbreviations: pre tr. = before arthroplasty, post tr. = after arthroplasty, diff. = difference, *d*_z_ = Cohen's effect size, *r* = Wilcoxon effect size.

**TABLE 6:** Arithmetic means of change in EEG bands power for patients in the knee arthroplasty group (motor imagery): Difference (post–pre arthroplasty). A positive value indicates an increase, a negative value indicates a decrease of absolute power after the procedure.

µV^2^	—	Theta	Low alpha	High alpha	Low beta	High beta
F3	Mean ± SD pre tr.	3.279 ± 0.738	6.229 ± 1.767	4.709 ± 1.820	2.228 ± 0.612	2.440 ± 0.521
—	Mean ± SD post tr.	4.207 ± 1.068	7.501 ± 2.382	4.620 ± 1.927	2.394 ± 0.614	2.675 ± 0.754
—	Post‐ pre diff. ± SD	0.929 ± 0.928	1.273 ± 1.605	−0.089 ± 0.713	0.166 ± 0.352	0.235 ± 0.393
—	diff. *p*‐value	<0.001	<0.001	0.012	<0.001	<0.001
—	diff. effect size	0.752 (*r*)	0.668 (*r*)	0.361 (*r*)	0.546 (*r*)	0.598 (*d*_z_)
Fz	Mean ± SD pre tr.	2.747 ± 0.500	7.950 ± 1.800	8.225 ± 2.675	2.356 ± 0.490	2.390 ± 0.594
—	Mean ± SD post tr.	3.150 ± 1.067	6.620 ± 2.052	9.368 ± 3.572	2.358 ± 0.620	2.293 ± 0.773
—	Post‐ pre diff. ± SD	0.403 ± 0.865	−1.330 ± 1.545	1.143 ± 1.632	0.001 ± 0.385	−0.098 ± 0.668
—	diff. *p*‐value	<0.001	<0.001	<0.001	0.163	0.153
—	diff. effect size	0.606 (*r*)	0.724 (*r*)	0.757 (*r*)	0.201 (*r*)	0.206 (*r*)
F4	Mean ± SD pre tr.	3.685 ± 1.145	7.973 ± 2.048	5.259 ± 2.131	2.290 ± 0.449	2.597 ± 0.916
—	Mean ± SD post tr.	4.748 ± 0.924	8.197 ± 2.370	5.011 ± 1.275	2.481 ± 0.713	2.809 ± 1.068
—	Post‐ pre diff. ± SD	1.064 ± 1.036	0.224 ± 1.574	−0.248 ± 1.338	0.191 ± 0.522	0.213 ± 0.801
—	diff. *p*‐value	<0.001	0.032	0.648	0.003	0.002
—	diff. effect size	1.027 (*d*_z_)	0.309 (*r*)	0.066 (*r*)	0.424 (*r*)	0.445 (*r*)
C3	Mean ± SD pre tr.	5.541 ± 1.444	5.723 ± 1.736	7.353 ± 2.998	1.829 ± 0.447	2.148 ± 0.389
—	Mean ± SD post tr.	5.285 ± 1.619	7.587 ± 2.577	7.206 ± 3.409	2.097 ± 0.467	2.576 ± 0.597
—	Post‐ pre diff. ± SD	−0.256 ± 0.785	1.865 ± 1.513	−0.147 ± 1.709	0.268 ± 0.373	0.429 ± 0.484
—	diff. *p*‐value	0.001	<0.001	0.554	<0.001	<0.001
—	diff. effect size	0.472 (*r*)	1.233 (*d*_z_)	0.086 (*d*_z_)	0.720 (*d*_z_)	0.768 (*r*)
Cz	Mean ± SD pre tr.	3.426 ± 0.936	7.026 ± 2.757	6.638 ± 3.221	2.003 ± 0.510	2.186 ± 0.489
—	Mean ± SD post tr.	3.280 ± 1.210	7.323 ± 3.097	7.576 ± 2.774	2.324 ± 0.533	1.989 ± 0.632
—	Post‐ pre diff. ± SD	−0.146 ± 0.629	0.298 ± 1.578	0.938 ± 1.550	0.321 ± 0.399	−0.196 ± 0.582
—	diff. *p*‐value	0.132	0.853	< 0.001	<0.001	<0.001
—	diff. effect size	0.540 (*r*)	0.027 (*r*)	0.577 (*r*)	0.806 (*d*_z_)	0.674 (*r*)
C4	Mean ± SD pre tr.	2.954 ± 0.981	7.041 ± 2.684	7.668 ± 3.777	2.002 ± 0.434	2.179 ± 0.700
—	Mean ± SD post tr.	3.671 ± 0.809	7.524 ± 2.898	5.478 ± 1.743	1.767 ± 0.379	1.932 ± 0.671
—	Post‐ pre diff. ± SD	0.717 ± 0.538	0.484 ± 1.108	−2.190 ± 3.442	−0.235 ± 0.364	−0.247 ± 0.302
—	diff. *p*‐value	<0.001	<0.001	<0.001	<0.001	<0.001
—	diff. effect size	0.795 (*r*)	0.552 (*r*)	0.587 (*r*)	0.547 (*r*)	0.695 (*r*)
P3	Mean ± SD pre tr.	3.541 ± 1.928	7.600 ± 3.553	11.089 ± 6.045	1.856 ± 0.521	1.956 ± 0.688
—	Mean ± SD post tr.	3.256 ± 1.314	9.011 ± 3.357	10.101 ± 3.382	1.751 ± 0.488	1.583 ± 0.395
—	Post‐ pre diff. ± SD	−0.285 ± 1.201	1.412 ± 1.650	−0.988 ± 6.521	−0.105 ± 0.377	−0.374 ± 0.609
—	diff. *p*‐value	<0.001	<0.001	0.488	0.003	<0.001
—	diff. effect size	0.479 (*d*_z_)	0.856 (*r*)	0.100 (*r*)	0.428 (*r*)	0.616 (*r*)
Pz	Mean ± SD pre tr.	2.954 ± 1.142	8.092 ± 3.725	11.950 ± 5.451	2.239 ± 0.669	1.576 ± 0.508
—	Mean ± SD post tr.	3.604 ± 0.936	10.064 ± 3.094	11.015 ± 6.661	2.153 ± 0.662	1.683 ± 0.544
—	Post‐ pre diff. ± SD	0.650 ± 0.765	1.972 ± 1.896	−0.935 ± 1.591	−0.086 ± 0.323	0.107 ± 0.271
—	diff. *p*‐value	<0.001	<0.001	<0.001	0.205	0.031
—	diff. effect size	0.717 (*d*_z_)	0.940 (*r*)	0.727 (*r*)	0.204 (*r*)	0.310 (*r*)
P4	Mean ± SD pre tr.	3.067 ± 1.673	7.777 ± 4.212	13.591 ± 6.185	2.476 ± 0.672	1.646 ± 0.571
—	Mean ± SD post tr.	3.526 ± 0.838	8.565 ± 3.972	11.660 ± 6.364	2.577 ± 0.690	2.084 ± 0.596
—	Post‐ pre diff. ± SD	0.459 ± 1.476	0.788 ± 1.418	−1.931 ± 2.721	0.101 ± 0.485	0.438 ± 0.427
—	diff. *p*‐value	<0.001	<0.001	<0.001	0.663	<0.001
—	diff. effect size	0.654 (*r*)	0.550 (*r*)	0.727 (*r*)	0.063 (*r*)	0.767 (*r*)

Abbreviations: pre tr. = before arthroplasty, post tr. = after arthroplasty, diff. = difference, *d*_z_ = Cohen's effect size, *r* = Wilcoxon effect size.

**TABLE 7:** Arithmetic means of change in EEG bands power for patients in the knee arthroplasty group (rest): Difference (post–pre arthroplasty). A positive value indicates an increase, a negative value indicates a decrease of absolute power after the procedure.

µV^2^		Theta	Low alpha	High alpha	Low beta	High beta
F3	Mean ± SD pre tr.	2.628 ± 0.607	6.109 ± 2.073	4.148 ± 1.626	2.093 ± 0.544	2.649 ± 0.536
—	Mean ± SD post tr.	3.090 ± 0.882	7.271 ± 3.017	4.138 ± 1.987	2.434 ± 0.693	2.970 ± 0.958
—	Post‐ pre diff. ± SD	0.463 ± 0.841	1.161 ± 1.774	−0.010 ± 1.046	0.340 ± 0.453	0.322 ± 0.630
—	diff. *p*‐value	<0.001	<0.001	0.362	<0.001	0.001
—	diff. effect size	0.534 (*r*)	0.688 (*r*)	0.132 (*r*)	0.665 (*r*)	0.399 (*r*)
Fz	Mean ± SD pre tr.	2.581 ± 0.567	5.377 ± 1.082	4.122 ± 1.526	2.150 ± 0.497	2.361 ± 0.559
—	Mean ± SD post tr.	2.805 ± 0.675	6.867 ± 2.882	5.279 ± 2.320	2.552 ± 0.596	2.856 ± 0.890
—	Post‐ pre diff. ± SD	0.224 ± 0.549	1.491 ± 2.967	1.157 ± 1.081	0.402 ± 0.443	0.494 ± 0.527
—	diff. *p*‐value	<0.001	0.019	<0.001	<0.001	<0.001
—	diff. effect size	0.542 (*r*)	0.338 (*r*)	0.790 (*r*)	0.754 (*r*)	0.938 (*d*_z_)
F4	Mean ± SD pre tr.	3.481 ± 0.800	5.163 ± 1.021	4.594 ± 2.201	2.179 ± 0.493	2.414 ± 0.885
—	Mean ± SD post tr.	4.611 ± 1.113	5.124 ± 1.723	5.194 ± 3.125	2.454 ± 0.748	2.574 ± 0.898
—	Post‐ pre diff. ± SD	1.130 ± 1.049	−0.039 ± 1.646	0.600 ± 1.336	0.275 ± 0.621	0.160 ± 0.505
—	diff. *p*‐value	<0.001	0.097	0.001	0.001	0.006
—	diff. effect size	1.077 (*d*_z_)	0.240 (*r*)	0.467 (*r*)	0.468 (*r*)	0.395 (*r*)
C3	Mean ± SD pre tr.	3.810 ± 1.603	6.019 ± 1.901	6.861 ± 2.529	2.024 ± 0.577	2.443 ± 0.528
—	Mean ± SD post tr.	4.568 ± 2.292	7.646 ± 2.649	6.637 ± 3.852	2.150 ± 0.644	2.801 ± 0.683
—	Post‐ pre diff. ± SD	0.758 ± 1.194	1.627 ± 1.787	−0.224 ± 2.378	0.126 ± 0.423	0.358 ± 0.452
—	diff. *p*‐value	<0.001	<0.001	0.518	0.036	<0.001
—	diff. effect size	0.697 (*r*)	0.665 (*r*)	0.094 (*d*_z_)	0.302 (*r*)	0.792 (*d*_z_)
Cz	Mean ± SD pre tr.	3.268 ± 1.106	7.274 ± 2.347	7.618 ± 3.045	1.999 ± 0.521	2.280 ± 0.468
—	Mean ± SD post tr.	3.818 ± 1.368	8.049 ± 3.178	6.269 ± 2.962	2.399 ± 0.673	2.912 ± 0.941
—	Post‐ pre diff. ± SD	0.549 ± 0.724	0.775 ± 1.563	−1.349 ± 1.879	0.399 ± 0.617	0.632 ± 0.827
—	diff. *p*‐value	<0.001	<0.001	<0.001	<0.001	<0.001
—	diff. effect size	0.692 (*r*)	0.532 (*r*)	0.621 (*r*)	0.610 (*r*)	0.666 (*r*)
C4	Mean ± SD pre tr.	3.391 ± 1.064	6.903 ± 2.651	7.948 ± 3.221	2.076 ± 0.372	2.418 ± 0.541
—	Mean ± SD post tr.	4.424 ± 1.111	8.663 ± 3.337	6.524 ± 5.417	2.059 ± 0.432	2.404 ± 0.642
—	Post‐ pre diff. ± SD	1.033 ± 1.040	1.760 ± 1.375	−1.425 ± 3.873	−0.017 ± 0.363	−0.013 ± 0.554
—	diff. *p*‐value	<0.001	<0.001	0.001	0.203	0.194
—	diff. effect size	0.993 (*d*_z_)	0.808 (*r*)	0.487 (*r*)	0.184 (*r*)	0.187 (*r*)
P3	Mean ± SD pre tr.	3.302 ± 1.574	7.706 ± 3.930	9.704 ± 3.850	1.819 ± 0.482	1.418 ± 0.351
—	Mean ± SD post tr.	3.520 ± 1.746	10.322 ± 3.614	10.408 ± 9.573	1.864 ± 0.497	1.517 ± 0.428
—	Post‐ pre diff. ± SD	0.218 ± 0.920	2.616 ± 1.556	0.704 ± 6.142	0.044 ± 0.407	0.099 ± 0.452
—	diff. *p*‐value	0.084	<0.001	0.335	0.010	0.135
—	diff. effect size	0.249 (*r*)	0.842 (*r*)	0.139 (*r*)	0.373 (*d*_z_)	0.220 (*r*)
Pz	Mean ± SD pre tr.	2.862 ± 1.093	7.692 ± 3.714	11.354 ± 6.156	2.128 ± 0.634	1.676 ± 0.447
—	Mean ± SD post tr.	3.316 ± 1.422	9.856 ± 3.853	9.561 ± 6.803	2.034 ± 0.722	4.262 ± 4.960
—	Post‐ pre diff. ± SD	0.454 ± 0.846	2.164 ± 1.868	−1.793 ± 2.719	−0.095 ± 0.470	2.586 ± 4.748
—	diff. *p*‐value	<0.001	<0.001	<0.001	0.004	0.177
—	diff. effect size	0.551 (*r*)	0.805 (*r*)	0.584 (*r*)	0.421 (*r*)	0.195 (*r*)
P4	Mean ± SD pre tr.	2.867 ± 0.892	7.846 ± 3.944	11.540 ± 4.793	2.431 ± 0.636	1.694 ± 0.413
—	Mean ± SD post tr.	3.273 ± 1.102	10.118 ± 4.245	10.153 ± 10.034	2.674 ± 0.729	2.159 ± 0.613
—	Post‐ pre diff. ± SD	0.406 ± 0.570	2.272 ± 2.210	−1.386 ± 5.696	0.244 ± 0.549	0.465 ± 0.533
—	diff. *p*‐value	<0.001	<0.001	<0.001	0.006	<0.001
—	diff. effect size	0.642 (*r*)	0.806 (*r*)	0.598 (*r*)	0.398 (*r*)	0.691 (*r*)

Abbreviations: pre tr. = before arthroplasty, post tr. = after arthroplasty, diff. = difference, *d*_z_ = Cohen's effect sizet, *r* = Wilcoxon effect size.

### 3.3 Clinical Improvement and EEG Band Power

Spearman correlations assessed relationships between ΔEEG absolute power (post‐ minus pre‐operative, µV^2^) and Δclinical scores (post‐ minus pre‐operative points): HHS (hip, *n* = 33) and KSS/OKS (knee, *n* = 48).

Hip arthroplasty group (HHS): Three moderate, significant correlations during the motor imagery task (MI):

↑ C4 high α power → HHS improvement (*r* = 0.438, *p* = 0.027)

↑ Fz high α power → HHS decline (*r* = ‐0.427, *p* = 0.046)

↑ P4 low α power → HHS decline (*r* = −0.453, *p* = 0.014)

No rest correlations with |*r*| > 0.4.

Knee arthroplasty group (KSS): One moderate significant correlation during rest:

↑ P4 low α power → KSS decline (*r* = −0.466, *p* = 0.001)

No MI correlations with |*r*| > 0.4.

(Full results: Tables 8 and 9.)

**TABLE 8:** Spearman's correlation for clinical improvement on the HHS scale and absolute change in EEG band power post hip arthroplasty for individual electrodes. *P*‐values are shown in parentheses.

—	ρ (*p*)	Theta	Low alpha	High alpha	Low beta	High beta
F3	Motor imagery task	−0.266 (0.135)	−0.104 (0.563)	0.011 (0.592)	−0.099 (0.582)	−0.024 (0.598)
—	Rest	0.206 (0.250)	0.379 (0.030)	−0.027 (0.883)	−0.110 (0.543)	−0.235 (0.189)
Fz	Motor imagery task	−0.108 (0.550)	−0.084 (0.643)	−0.427 (0.046)	−0.128 (0.479)	0.075 (0.680)
—	Rest	0.087 (0.632)	0.226 (0.206)	−0.233 (0.192)	−0.112 (0.536)	0.069 (0.704)
F4	Motor imagery task	−0.222 (0.214)	0.043 (0.813)	−0.016 (0.390)	−0.064 (0.724)	0.010 (0.495)
—	Rest	0.006 (0.479)	0.121 (0.502)	−0.227 (0.204)	0.167 (0.353)	0.119 (0.509)
C3	Motor imagery task	0.150 (0.405)	−0.134 (0.457)	−0.099 (0.584)	0.125 (0.489)	−0.087 (0.630)
—	Rest	0.125 (0.489)	0.015 (0.935)	0.199 (0.267)	0.139 (0.439)	−0.085 (0.639)
Cz	Motor imagery task	−0.013 (0.495)	−0.080 (0.658)	−0.135 (0.455)	−0.135 (0.452)	0.016 (0.391)
—	Rest	0.186 (0.299)	0.191 (0.288)	0.019 (0.915)	−0.102 (0.571)	−0.082 (0.649)
C4	Motor imagery task	−0.151 (0.402)	−0.084 (0.641)	0.438 (0.027)	−0.123 (0.497)	0.200 (0.266)
—	Rest	−0.093 (0.605)	−0.026 (0.885)	0.306 (0.083)	−0.301 (0.089)	0.039 (0.830)
P3	Motor imagery task	0.085 (0.637)	−0.138 (0.442)	−0.176 (0.328)	0.199 (0.267)	−0.033 (0.587)
—	Rest	0.014 (0.397)	−0.113 (0.530)	0.017 (0.927)	−0.083 (0.647)	0.018 (0.292)
Pz	Motor imagery task	−0.092 (0.611)	−0.090 (0.618)	0.192 (0.285)	0.117 (0.517)	−0.147 (0.414)
—	Rest	0.036 (0.842)	−0.123 (0.495)	0.185 (0.302)	0.161 (0.372)	0.009 (0.960)
P4	Motor imagery task	−0.011 (0.592)	−0.453 (0.014)	0.133 (0.461)	0.064 (0.724)	−0.025 (0.889)
—	Rest	−0.168 (0.350)	−0.052 (0.772)	−0.052 (0.775)	−0.098 (0.589)	0.167 (0.352)

**TABLE 9:** Spearman's correlation for clinical improvement on the KSS scale and absolute change in EEG band power post knee arthroplasty for individual electrodes. *P*‐values are given in parentheses.

—	ρ (*p*)	Theta	Low alpha	High alpha	Low beta	High beta
F3	Motor imagery task	−0.136 (0.357)	−0.003 (0.983)	0.173 (0.241)	−0.144 (0.329)	−0.301 (0.038)
—	Rest	0.102 (0.492)	−0.014 (0.922)	0.188 (0.201)	−0.072 (0.628)	−0.302 (0.037)
Fz	Motor imagery task	−0.184 (0.212)	0.065 (0.659)	−0.342 (0.017)	−0.064 (0.667)	−0.171 (0.245)
—	Rest	0.074 (0.615)	0.232 (0.113)	0.056 (0.707)	−0.299 (0.039)	−0.229 (0.118)
F4	Motor imagery task	−0.347 (0.016)	0.071 (0.631)	−0.111 (0.455)	−0.289 (0.046)	−0.178 (0.227)
—	Rest	−0.292 (0.044)	0.239 (0.102)	−0.020 (0.893)	0.125 (0.397)	−0.144 (0.331)
C3	Motor imagery task	−0.008 (0.956)	0.126 (0.394)	0.062 (0.673)	−0.282 (0.052)	−0.216 (0.140)
—	Rest	−0.056 (0.704)	0.053 (0.723)	0.141 (0.340)	−0.194 (0.187)	−0.332 (0.021)
Cz	Motor imagery task	0.266 (0.067)	−0.017 (0.910)	0.053 (0.721)	−0.261 (0.073)	0.237 (0.105)
—	Rest	−0.145 (0.324)	0.142 (0.335)	0.226 (0.122)	−0.311 (0.032)	−0.291 (0.045)
C4	Motor imagery task	−0.166 (0.259)	−0.157 (0.287)	0.310 (0.032)	0.202 (0.168)	0.247 (0.091)
—	Rest	−0.351 (0.014)	−0.248 (0.089)	0.255 (0.081)	0.194 (0.186)	0.239 (0.101)
P3	Motor imagery task	−0.075 (0.610)	−0.204 (0.165)	0.146 (0.323)	0.040 (0.785)	0.231 (0.114)
—	Rest	−0.392 (0.006)	−0.376 (0.008)	0.163 (0.268)	−0.163 (0.267)	0.116 (0.433)
Pz	Motor imagery task	−0.292 (0.044)	−0.228 (0.119)	0.092 (0.534)	0.112 (0.447)	−0.049 (0.743)
—	Rest	−0.135 (0.360)	−0.262 (0.072)	0.121 (0.414)	−0.037 (0.802)	0.043 (0.771)
P4	Motor imagery task	−0.334 (0.020)	−0.392 (0.006)	0.230 (0.117)	−0.201 (0.171)	−0.128 (0.385)
—	Rest	−0.231 (0.114)	−0.466 (0.001)	0.075 (0.613)	−0.292 (0.044)	−0.158 (0.282)

## 4 Discussion

In this pre–post EEG study of patients undergoing total hip or knee arthroplasty for end‐stage osteoarthritis, we observed extensive and clinically relevant changes in cortical oscillatory activity three months after surgery. The most consistent effect, present irrespective of joint and condition (rest and stair‐climbing motor imagery), was an increase in low alpha band power over frontal, central, and parietal regions, often with medium to large effect sizes. Theta and beta band power also showed postoperative increases in several regions of interest, whereas high alpha power followed a mixed pattern with both increases and decreases depending on electrode location and task.

In parallel, we noted marked improvement in joint‐specific clinical scores (HHS for hip, KSS for knee), confirming the effectiveness of arthroplasty and early rehabilitation in restoring function. When changes in EEG band power were related to changes in clinical scores, only a small number of moderate associations emerged, predominantly in the hip cohort: greater HHS improvement was associated with increased high alpha power at C4, decreased high alpha power at Fz, and decreased low alpha power at P4 during motor imagery. In the knee cohort, a single moderate association indicated that increased low alpha power at P4 at rest co‐occurred with smaller gains in KSS.

Taken together, these findings indicate that total hip and knee arthroplasty are accompanied by early cortical reorganization detectable with quantitative EEG, and that interventions on weight‐bearing joints, together with rehabilitation, modify not only peripheral biomechanics but also central sensorimotor processing. Two aspects appear novel: first, the demonstration of joint‐ and region‐specific changes in resting and motor‐imagery EEG band power in a relatively large, clinically realistic arthroplasty cohort; and second, the initial evidence that specific post‐operative alpha band changes relate to functional gains, especially after hip replacement.

The observed postoperative increase in low alpha power extends previous EEG research in osteoarthritis and chronic knee pain, where cross‐sectional studies reported altered resting‐state oscillations (theta and alpha changes) in comparison with pain‐free controls, interpreted in terms of thalamocortical dysrhythmia and impaired cortical inhibition. In particular, greater low‐frequency (delta, theta) power and alterations in alpha/beta power over sensorimotor regions have been linked to more severe disease, higher pain, and worse motor performance (Simis et al. 2022; Mathew et al. 2024). In contrast to these cross‐sectional designs, our within‐subject pre–post approach after “structural correction” of the joint and standardized rehabilitation suggests that the prominent increase in low alpha across frontocentral and parietal regions may reflect a shift from a pain‐dominated, maladaptive oscillatory state toward a more regulated sensorimotor configuration. While earlier longitudinal rehabilitation work in knee osteoarthritis reported only trends toward increased beta and decreased alpha power after non‐surgical interventions, without statistically significant group‐level EEG changes, our findings imply that the magnitude of peripheral change and functional recovery may be critical determinants of detectable cortical reorganization (Van Cauwenbergh et al. 2026).

The pattern of alpha and beta modulation is also consistent with the broader literature on EEG correlates of movement and motor imagery, in which sensorimotor alpha/beta rhythms typically show event‐related desynchronization during actual or imagined movement and relative resynchronization at rest, and are therefore interpreted as markers of cortical idling and inhibitory control (Yuan et al. 2010; Magosso et al. 2019). In the present study, we analyzed absolute band power in 10 s epochs rather than time‐locked ERD/ERS measures, limiting direct comparison with classical motor imagery paradigms; nonetheless, the robust postoperative increase in low alpha during both rest and motor imagery, particularly over central and parietal sites, may indicate improved capacity of sensorimotor networks to down‐regulate background excitability and allocate resources efficiently during imagined functional tasks.

Our results also converge conceptually with evidence of central reorganization following ACL injury and reconstruction (Baumeister et al. 2008; Baumeister et al. 2011). Multiple EEG and imaging studies have shown altered activation and connectivity within primary sensorimotor, premotor and cerebellar networks after ACL injury, interpreted as compensatory adaptations to disrupted ligament proprioception and altered sensory input (Grooms et al. 2015; Miao et al. 2017; Needle et al. 2017; Stańczak et al. 2025).

Similar to those observations, the present work indicates that chronic degenerative joint disease and its surgical treatment are associated with measurable modifications in cortical oscillatory patterns. However, whereas ACL studies often focus on task‐related activation during force control or joint position tasks in younger, athletic populations, the current study provides complementary evidence in older osteoarthritis patients performing a functionally relevant stair‐climbing motor imagery task.

Direct qEEG studies after hip or knee arthroplasty remain scarce. Early work in THA described postoperative changes in brain bioelectrical activity without systematic band‐specific analysis or linkage to function (Barabash et al. 2000). More recent neurorehabilitation studies in TKA have emphasized neuromodulatory interventions (e.g., end‐effector gait robots, combined motor imagery and active exercise) without detailed spectral EEG characterization (Lu et al. 2025; Hyung et al. 2025). By integrating HHS and KSS with quantitative EEG during both rest and motor imagery, our findings help to fill this gap and provide a more direct link between cortical oscillations and functional status in the early post‐arthroplasty period.

A central result is the consistent increase in low alpha power in both cohorts. Low alpha oscillations over sensorimotor and parietal regions have been associated with cortical inhibition, top‐down control of sensory processing, and efficient information gating. In chronic pain populations, impaired alpha modulation has been linked to central sensitization, abnormal thalamic gating, and heightened nociceptive processing (Simis et al. 2022; Rocha et al. 2020). In this context, the clinical success of arthroplasty and rehabilitation may partially normalize inhibitory mechanisms within sensorimotor networks, thereby reducing the central component of pain and supporting more economical motor planning during functional tasks such as stair climbing ^34‐36.^

At the same time, postoperative increases in theta and high beta power, especially over central and parietal areas, may reflect processes of updating the internal model of the limb after prosthesis implantation. Theta activity is commonly related to working memory and multimodal integration, while beta rhythms are associated with maintaining the current motor set and cortico‐muscular coupling. [34–36] In the setting of a new prosthetic joint, increased theta and beta power may therefore index greater engagement of networks responsible for monitoring joint position, load, and stability.

The EEG–clinical correlations point to important region‐, task‐, and joint‐specific effects. In TKA patients, the negative relationship between increased low alpha power at P4 at rest and KSS gain may suggest that excessive parietal inhibition hampers optimal adaptation in some individuals, given the role of parietal regions in visuospatial integration and body‐schema representation. Conversely, in THA, the positive association between increased high alpha power at C4 during motor imagery and HHS improvement implies that, in a stabilized joint, local alpha oscillations may support more efficient motor planning and imagery. However, the small number and inconsistency of correlations underscore that cortical oscillations represent only one component of the complex recovery process after arthroplasty.

From a clinical perspective, the present findings reinforce the notion that major orthopedic interventions should be conceptualized not only as mechanical repairs but also as triggers of central neuroplasticity. Quantitative EEG offers a non‐invasive, relatively accessible window into this cortical adaptation process. The demonstration that significant band‐power changes occur as early as three months post‐arthroplasty suggests that EEG could be used to monitor the trajectory of central recovery, identify patients whose cortical responses are delayed or atypical, and tailor rehabilitation intensity and content accordingly. For example, individuals with persistently elevated frontal beta power or insufficient normalization of low alpha rhythms might benefit from targeted interventions aimed at modulating these oscillations (Simis et al. 2022; Mathew et al. 2024). Evidence from neurofeedback studies in pain indicates that increasing alpha power can reduce pain ratings and alter pain‐evoked potentials, supporting the feasibility of such approaches (Birch et al. 2022; Hesam‐Shariati et al. 2022).

Moreover, the robust EEG responses during stair‐climbing motor imagery confirm that older arthroplasty patients can effectively engage motor‐related cortical circuits via mental practice alone, which supports the integration of motor imagery and brain–computer interface paradigms into rehabilitation after total knee and hip arthroplasty, particularly in the early postoperative phase when pain and weight‐bearing restrictions limit active training. Finally, the weak associations between EEG changes and HHS/KSS highlight the influence of moderators such as pain chronicity, psychosocial factors (e.g., kinesiophobia), muscle strength, and residual joint biomechanics, and argue for interpreting our findings within a biopsychosocial model of post‐arthroplasty recovery. Nonetheless, the systematic, band‐specific qEEG changes after hip and knee replacement support the central thesis that successful arthroplasty is accompanied by measurable cortical reorganization, which may in the future inform prognostic stratification and the design of personalized rehabilitation and neuromodulatory interventions.

## 5 Study Limitations

### 5.1 Spatial Resolution

EEG lacks the spatial precision necessary for differentiating activation at the level of individual gyri or sulci and cannot reliably capture subcortical activity, reflecting signals mainly from the outer 1 cm of cortex  (Wieser et al. 2010). This constrains accurate assessment of lower limb motor representations, which lie deeper (1–4 cm) and medially within the interhemispheric fissure with vertically oriented cortical surfaces, making left–right leg discrimination difficult  (Kline et al. 2021; Boord et al. 2010).

### 5.2 Temporal scope

The 3‐month follow‐up captures only early neuroplastic adaptation after arthroplasty. Rehabilitation and cortical reorganization often continue for 12–24 months; thus, findings represent a brief snapshot and may reflect transient rather than lasting neural changes. Extended longitudinal designs (6–24 months) are needed to clarify these dynamics.

### 5.3 Outcome Asymmetry

Knee patients were evaluated with KSS and OKS, whereas hip patients were assessed using HHS, limiting direct comparison of subjective recovery between groups.

### 5.4 Motor Task Specificity

The single motor imagery task (stair climbing) limits generalizability, as real‐life rehabilitation engages diverse motor networks (walking, sit‐to‐stand, balance). Including multiple or executed tasks (e.g., EMG‐EEG co‐registration) would improve ecological validity.

### 5.5 Clinical Uncertainty

Few significant EEG‐function correlations (three in hip, one in the knee cohort) suggest EEG reflects only part of the multifactorial recovery process. The predictive or biomarker value of EEG remains uncertain. Future EEG‐fMRI studies may elucidate the neural mechanisms underlying joint‐specific differences.

### 5.6 Methodological Constraints

The lack of a control group and single‐epoch power estimation introduce variance, though the pre–post design supports internal validity. No correction for multiple comparisons was applied, so marginal *p*‐values should be interpreted cautiously.

## Author Contributions

**Pawel Piotr Dobrakowski**: conceptualization, writing – original draft, investigation, methodology.

## Funding

The authors have nothing to report.

## Ethics Statement

The study involving human participants was reviewed and approved by the Ethics Committee of the Academy of Physical Education in Katowice.

## Consent

Written informed consent to participate in this study was provided by the participants.

## Conflicts of Interest

Dr Jaroslaw Szyszka works in Opole Rehabilitation Center, Korfantów

## Data Availability

The data that support the findings of this study are available on request from the corresponding author. The data are not publicly available due to privacy of patients.
